# Healthcare-associated pneumonia among hospitalized patients in a Korean tertiary hospital

**DOI:** 10.1186/1471-2334-11-61

**Published:** 2011-03-11

**Authors:** Ji Ye Jung, Moo Suk Park, Young Sam Kim, Byung Hoon Park, Se Kyu Kim, Joon Chang, Young Ae Kang

**Affiliations:** 1 Division of Pulmonology and Critical Care Medicine, Department of Internal Medicine, Institute of Chest Disease, Yonsei University College of Medicine, Seoul, Republic of Korea

## Abstract

**Background:**

Healthcare-associated pneumonia (HCAP) has more similarities to nosocomial pneumonia than to community-acquired pneumonia (CAP). However, there have only been a few epidemiological studies of HCAP in South Korea. We aimed to determine the differences between HCAP and CAP in terms of clinical features, pathogens, and outcomes, and to clarify approaches for initial antibiotic management.

**Methods:**

We conducted a retrospective, observational study of 527 patients with HCAP or CAP who were hospitalized at Severance Hospital in South Korea between January and December 2008.

**Results:**

Of these patients, 231 (43.8%) had HCAP, and 296 (56.2%) had CAP. Potentially drug-resistant (PDR) bacteria were more frequently isolated in HCAP than CAP (12.6% vs. 4.7%; *P *= 0.001), especially in the low-risk group of the PSI classes (41.2% vs. 13.9%; *P *= 0.027). In-hospital mortality was higher for HCAP than CAP patients (28.1% vs. 10.8%, *P *< 0.001), especially in the low-risk group of PSI classes (16.4% vs. 3.1%; *P *= 0.001). Moreover, tube feeding and prior hospitalization with antibiotic treatment within 90 days of pneumonia onset were significant risk factors for PDR pathogens, with odds ratios of 14.94 (95% CI 4.62-48.31; *P *< 0.001) and 2.68 (95% CI 1.32-5.46; *P *= 0.007), respectively.

**Conclusions:**

For HCAP patients with different backgrounds, various pathogens and antibiotic resistance of should be considered, and careful selection of patients requiring broad-spectrum antibiotics is important when physicians start initial antibiotic treatments.

## Background

Pneumonia has traditionally been classified as either community- or hospital- acquired, based on the patient's location when the infection was acquired. However, an increasing number of out-of-hospital services, such as nursing homes, outpatient parenteral therapy, hemodialysis clinics, and domiciliary care, create a class of patients who do not truly reside in the "community."

Previous studies have suggested that nursing home-acquired pneumonia or pneumonia in long-term care facilities should be considered separately from community-acquired pneumonia (CAP) [1-3]. Infections occurring in these patients show a more similar epidemiological pattern to hospital-acquired pneumonia (HAP) than to CAP [1-3]. Different epidemiological patterns from CAP require a distinct targeted therapeutic approach, especially against multidrug-resistant pathogens [4,5]. Thus, since 2000, the newly published CAP guidelines have recommended management specific to this type of pneumonia, and considered it to be a separate entity from CAP [6-8].

In 2005, the American Thoracic Society (ATS)/Infectious Diseases Society of America (IDSA) documented healthcare-associated pneumonia (HCAP) as a new category of pneumonia [9]. However, only a few studies on HCAP have included patients who met the criteria of the 2005 ATS/IDSA guidelines[10]. Previous HCAP studies have revealed a diverse composition of participants because this new HCAP category includes various criteria for heterogeneous conditions, such as nursing home residence, previous antibiotic therapy, or regular attendance at a dialysis clinic [11-13]. Since the criteria for defining HCAP have not been standardized between these studies, and due to the existence of geographically different etiologies, more data are required for a better characterization of unified HCAP and for redefining HCAP.

In South Korea, there are limited data and no therapeutic guidelines focusing on HCAP [14]. Considering the relatively small proportion of long-term care facilities and the different antibiotic resistance patterns of the microorganisms in CAP, the clinical composition, causative pathogens, and clinical outcomes of HCAP in South Korea could be different from those in other countries. Therefore, a study evaluating HCAP characteristics and clinical outcomes in South Korea is needed.

The aim of this study was to categorize patients according to the 2005 ATS/IDSA guidelines, to determine differences in baseline characteristics, pathogens, and clinical outcomes between patients with HCAP and CAP in a university teaching hospital in South Korea, and to clarify approaches for initial antibiotic management.

## Methods

### Study design and subjects

We conducted a retrospective observational study of 527 patients with CAP or HCAP who were hospitalized at Severance Hospital (a 2,000-bed university tertiary referral hospital in Seoul, South Korea) between January 1 and December 31, 2008. Patients were classified into either a CAP or HCAP group, according to the 2005 ATS/IDSA guidelines. We compared baseline characteristics, and identified pathogens, antibiotics regimens, and clinical outcomes between the two groups. The study protocol was approved by the Ethical Review Committee of Severance Hospital.

### Definitions

Pneumonia was defined as the presence of a new infiltrate on the chest radiography plus at least one of the following: fever (temperature ≥ 38.0°C) or hypothermia (temperature < 35.0°C), new cough with or without sputum production, pleuritic chest pain, dyspnea, or altered breath sounds on auscultation [15].

HCAP included any patient who fulfilled any of the following: (1) hospitalization in an acute care hospital for two or more days within 90 days of the infection; (2) residence in a nursing home or long-term care facility; (3) infusion therapy, such as intravenous antibiotic therapy, chemotherapy or wound care, within 30 days of a current infection; (4) regular attendance at a dialysis clinic, including hemodialysis and peritoneal dialysis [9]. CAP included any patient with pneumonia who did not meet the HCAP criteria.

Patients were defined as being immunosuppressed if they fulfilled at least one of the following criteria: (1) daily administration of systemic corticosteroids (at least 15 mg of prednisone per day for more than one month or combination therapy with low dose corticosteroids and other immunosuppressants including azathioprine, mycophenolate, methotrexate, cyclosporine, or cyclophosphamide) (2) seropositivity for human immunodeficiency virus; (3) receipt of either a solid organ or bone marrow transplant; (4) treatment with radiation therapy or chemotherapy for an underlying malignancy during the 6 months prior to hospital admission; or (5) underlying acquired immune deficiency disorder [11,13].

In this study, potentially drug resistant (PDR) pathogens included methicillin-resistant *Staphylococcus aureus *(MRSA), *Pseudomonas aeruginosa*, *Acinetobacter baumannii*, *Stenotrophomonas maltophilia*, and extended spectrum *β-*lactamase (ESBL)-producing *Enterobacteriaceae*, based on previous reports showing problematic clinical outcomes for infections caused by these pathogens [16,17].

Inappropriate antibiotic therapy was defined as when any initially prescribed antibiotics were not active against the identified pathogens, based on *in vitro *susceptibility testing [10,12].

The definition of early treatment failure was clinical deterioration within 72 hours of starting treatment, such as a lack of response or worsening of fever, respiratory condition, and/or radiographic status, requiring mechanical ventilation or aggressive fluid resuscitation or vasopressors, or death [12,18,19].

The severity of pneumonia was evaluated and categorized using the validated prediction rule and pneumonia severity index (PSI) scores: low, class I to III; intermediate, class IV; high, class V [20,21].

### Antibiotic treatment

Antibiotic therapy was initiated in basic accordance with the ATS/IDSA guidelines (8,9), but the detailed antibiotic regimen complied with the attending physician's choice taking into consideration patient risk factors and the severity of the disease. Empirical antibiotic therapy was modulated after the pathogen was identified according to the susceptibility test. However, the antibiotic therapy was changed or extended according to the attending physician's decision for patients in whom the pathogen was not identified or whose clinical condition deteriorated.

### Microbiological studies

Pathogens in samples obtained from sputum, blood, or other samples were investigated using standard microbiological procedures. Blood cultures were accepted as an etiological diagnosis if no other source could be identified for the positive blood culture. Sputum samples were cultured in a semi-quantitative manner, and an etiological diagnosis was established when a predominant microorganism was isolated from group 4 or 5 sputum, according to Murray and Washington's grading system [22]. A rapid immunochromatographic assay was used for detecting the *Streptococcus pneumoniae *antigen (BinaxNOW^® ^*S. pneumoniae *Test; Binax Inc., Scarborough, ME, USA) and *Legionella pneumophila *serogroup I antigen (BinaxNOW^® ^*Legionella *Test; Binax Inc., Scarborough, ME, USA) in urine. Antibodies against atypical pathogens (*Mycoplasma pneumoniae*) were detected by microparticle agglutination assay (MAG). Cases that did not meet any of these criteria were considered to be pneumonia of unknown etiology. The antibiotic sensitivity of all isolates was determined using a disc diffusion method, according to the Clinical and Laboratory Standards Institute guidelines [23].

### Statistical analyses

Categorical variables were analyzed using the χ^2 ^test or Fisher's exact test, and continuous variables were analyzed using Student's *t*-test or Mann-Whitney U test. After testing the distribution of continuous variables, normally distributed variables were presented as mean ± standard deviation (SD) and non-normally distributed variables were presented as median (interquartile range, [IQRs]). Multivariate analysis was performed using a logistic regression model to estimate risk factors for occurrence of PDR pathogens, which was presented with the odds ratio (OR, 95% confidence intervals, CI). Potential candidate variables were those with *P *< 0.05 in univariate analyses, and the multi-collinearity of variables was checked. All tests were two-sided and a *P-*value < 0.05 was deemed to be statistically significant. SPSS 18.0 (SPSS, Chicago, IL, USA) was used for all statistical analyses.

## Results

### Patient characteristics

Of the 527 patients, 231 (43.8%) were classified as HCAP and 296 (56.2%) as CAP. The baseline and clinical characteristics of the patients with HCAP and CAP are shown in Table 1. Of the 231 HCAP patients, 170 (73.6%) had been hospitalized for two or more days within 90 days of pneumonia, 150 (64.9%) received intravenous antibiotic therapy/chemotherapy/wound care within 30 days of pneumonia onset, 24 (10.4%) attended a hemodialysis clinic, and 21 (9.1%) resided in a nursing home or extended care facility (data not shown).

**Table 1:** Baseline Characteristics of Patients with HCAP and CAP^a^

Baseline Characteristics	HCAP (n = 231)	CAP (n = 296)	*P-*value
Age (year)	63.5 ± 13.1	65.4 ± 15.7	0.148
Male	162 (70.1)	194 (65.5)	0.264
Female	69 (29.9)	102 (34.5)	
Underlying diseases			
Diabetes Mellitus	52 (22.5)	59 (19.9)	0.471
Chronic lung disease^b^	22 (9.5)	56 (18.9)	0.003
Central nervous system disorders	26 (11.3)	47 (15.9)	0.127
Renal disease	31 (13.4)	27 (9.1)	0.118
Hypertension	93 (40.3)	125 (42.2)	0.649
Cardiovascular disease	19 (8.2)	62 (20.9)	<0.001
Liver disease	12 (5.2)	16 (5.4)	0.915
Rheumatologic disease	9 (3.9)	18 (6.1)	0.259
Malignancy	156 (67.5)	58 (19.6)	<0.001
Solid organ malignancy	133 (57.6)	42 (14.2)	<0.001
Hematologic malignancy	26 (11.3)	17 (5.7)	0.022
Clinical parameters			
Confusion	35 (15.2)	34 (11.5)	0.216
Septic shock at onset	57 (24.7)	46 (15.5)	0.009
PaO_2 _< 60 mmHg, SpO_2 _< 90%, or PaO_2_/FiO2 < 300	79 (34.2)	96 (32.4)	0.669
Pleural effusion	43 (18.6)	49 (16.6)	0.536
Acute renal failure at onset	32 (13.9)	37 (12.5)	0.648
Laboratory findings			
pH < 7.35	10 (4.3)	23 (7.8)	0.106
Hematocrit < 30%	87 (37.7)	51 (17.2)	<0.001
Glucose > 250 mg/dL	20 (8.7)	21 (7.1)	0.506
Blood urea nitrogen > 30 mg/dL	67 (29.0)	49 (16.6)	0.001
Leukocytes (×10^3^/μl)	12790 ± 13603	13175 ± 7519	0.700
C-reactive protein (mg/dL)	15.4 ± 11.2	13.1 ± 10.0	0.017
Erythrocyte Sedimentation Rate (mm)	83.2 ± 33.8	71.4 ± 35.7	<0.001
Eastern Cooperative Oncology Group (ECOG)	1.7 ± 0.9	1.2 ± 0.9	<0.001
Prior antibiotics use	97 (42.0)	6 (1.0)	<0.001
Immunosuppressed^c^	142 (61.5)	39 (13.2)	<0.001
Pneumonia Severity Index Risk Classes	113.7 ± 32.3	92.2 ± 33.6	<0.001
Low (I-III)	55 (23.8)	159 (53.7)	
Intermediate (IV)	115 (49.8)	99 (33.4)	<0.001
High (V)	61 (26.4)	38 (12.8)	

^a ^Data are presented as numbers (percentages) unless otherwise indicated. Plus-minus values are means ± standard deviation.

^b ^Chronic lung disease includes asthma, COPD, and structural lung diseases, such as bronchiectasis and interstitial lung disease

^c ^Immunosuppression includes the following: (1) daily administration of systemic corticosteroids (at least 15 mg of prednisone per day for more than one month or combination therapy with low dose corticosteroids and other immunosuppressants including azathioprine, mycophenolate, methotrexate, cyclosporine, or cyclophosphamide); (2) seropositivity for human immunodeficiency virus; (3) received either a solid organ transplant or bone marrow transplant; (4) treated with radiation therapy or chemotherapy for an underlying malignancy during the 6 months prior to hospital admission; (5) an underlying acquired immune deficiency disorder

HCAP healthcare-associated pneumonia

CAP community-acquired pneumonia

### Pathogen distribution

Table 2 shows the distribution of causative organisms. The numbers of sputum samples evaluated for pathogens were 203 (87.9%) in HCAP patients and 266 (89.9%) in CAP patients, and those of blood samples were 230 (99.6%) in HCAP patients and 289 (97.6%) in CAP patients (data not shown). An etiological diagnosis was established in 79 HCAP (34.2%) and 83 CAP patients (29.1%) (*P *= 0.206). *S. pneumoniae *was the most frequently isolated pathogen in CAP patients, while *Klebsiella pneumoniae *was most frequently isolated in HCAP patients. More PDR pathogens were observed in patients with HCAP.

**Table 2:** Pathogen Distribution in Patients with HCAP and CAP^a^

Microbes	HCAP (*n *= 231)	CAP (*n *= 296)	*P-*value
**Pathogen identified**	79 (34.2)	83 (29.1)	0.206
**Gram-positive pathogens**	34 (14.7)	54 (18.2)	0.282
MRSA	9 (3.9)	6 (2.0)	
MSSA	4 (1.7)	9 (3.0)	
*Streptococcus pneumoniae*	11 (4.8)	33 (11.1)	
*Streptococcus *other	4 (1.7)	3 (1.0)	
*Enterococcus *species	0 (0)	1 (0.3)	
Others ^b^	6 (2.6)	3 (1.0)	
**Gram-negative pathogens**	46 (19.9)	28 (9.5)	0.001
*Pseudomonas aeruginosa*	10 (4.3)	5 (1.7)	
*Escherichia coli*	1 (0.4)	0 (0)	
*Haemophilus influenzae*	2 (0.9)	3 (1.0)	
*Klebsiella pneumoniae*	23 (10.0)	13 (4.4)	
ESBL producing ^c^	9/23 (39.1)	3/13 (23.1)	
*Enterobacter *species	4 (1.7)	1 (0.3)	
*Acinetobacter baumannii*	1 (0.4)	0 (0)	
*Stenotrophomonas maltophilia*	1 (0.4)	1 (0.3)	
Others ^d ^	4 (1.7)	5 (1.7)	
Anaerobes ^e^	1 (0.4)	0 (0)	
Atypical pathogens ^f^	0 (0)	3 (1.0)	
Fungal pathogens^g^	1 (0.4)	1 (0.3)	
PDR pathogens	29 (12.6)	14 (4.7)	0.001

^a ^Data are presented as numbers (percentages) unless otherwise indicated.

^b ^Coagulase-negative *Staphylococci *species

^c ^Number of patients/total number of patients in whom *Klebsiella pneumonia *was identified (percentages)

^d ^*Moraxella catarrhalis *(1 in HCAP and 3 in CAP), *Serratia marcescens *(2 in HCAP and 2 in CAP), C*itrobacter freundii *(1 in HCAP)

^e ^*Bacteroides fragilis*

^f ^*Mycoplasma pneumonia (*3 in CAP)

^g ^*Aspergillus *species

HCAP healthcare-associated pneumonia

CAP community-acquired pneumonia

MRSA methicillin-resistant *Staphylococcus aureus*

MSSA methicillin-sensitive *Staphylococcus aureus*

ESBL extended-spectrum β-lactamase

PDR potentially drug-resistant

### Antibiotic treatment and clinical outcomes

Initial antibiotic treatments and outcomes for patients with HCAP and CAP are shown in Table 3. Inappropriate antibiotic therapy tended to be administered more frequently to HCAP patients than CAP patients, although the difference was not significant. Patients in both groups received combination therapy more than monotherapy. The in-hospital mortality and early treatment failure rates were significantly higher in HCAP patients than CAP patients. Patients with HCAP stayed in the hospital longer and showed a more frequent need for mechanical ventilation than patients with CAP.

**Table 3:** Antibiotic Treatments and Clinical Outcomes in Patients with HCAP and CAP^a^

Treatment	HCAP (*n *= 231)	CAP (*n *= 296)	*P-*value
**Inappropriate antibiotic therapy**^b^	27/73 (37.0)	19/78 (24.4)	0.092
**Monotherapy**	30 (13.0)	53 (17.9)	0.124
Amino-penicillins	3 (1.3)	5 (1.7)	-
Cephalosporin	5 (2.2)	8 (2.7)	-
Antipseudomonal penicillins	6 (2.6)	6 (2.0)	-
Fluroquinolone	13 (5.6)	34 (11.5)	-
Carbapenem	3 (1.3)	0 (0)	-
**Combination therapy**	201 (87.0)	243 (82.1)	0.124
*β*-lactams + fluoroquinolone	4 (1.7)	8 (2.7)	-
*β*-lactams + macrolide	36 (15.6)	112 (37.8)	<0.001
*β*-lactams + clindamycin	10 (4.3)	14 (4.7)	-
*β*-lactams + aminoglycoside	6 (2.6)	1 (0.3)	-
Fluoroquinolone + clindamycin	13 (5.6)	12 (4.1)	-
Antipseudomonal *β*-lactams + fluroquinolone	63 (27.3)	48 (16.2)	0.001
Antipseudomonal *β*-lactams + macrolide	0 (0)	1 (0.3)	-
Antipseudomonal *β*-lactams + clindamycin	6 (2.6)	3 (1.0)	-
Antipseudomonal *β*-lactams + aminoglycoside	8 (3.5)	2 (0.7)	-
Other combination therapy^c^	55 (23.8)	42 (14.2)	0.002
**Clinical outcomes**			
In-hospital mortality	65 (28.1)	32 (10.8)	<0.001
Early treatment failure^d^	56 (24.2)	37 (12.5)	<0.001
ICU admission	49 (21.2)	37 (12.5)	0.007
ICU mortality	31 (13.4)	21 (7.1)	0.016
Need for mechanical ventilation	47 (20.3)	39 (13.2)	0.027
Duration of hospital stay (days)	18.6 ± 19.1	12.9 ± 13.1	<0.001

^a ^Data are presented as numbers (percentages) unless otherwise indicated. Plus-minus values are means ± standard deviation.

^b ^Number of patients/total number of patients whose causative pathogens and antibiotic sensitivity test results are known (percentages). Results of antibiotics sensitivity test were not available in six patients with HCAP and five patients with CAP.

^c ^Others contain combination therapy of three or more drugs, including aminopenicillins, cephalosporin, antipseudomonal penicillin, aminglycoside, macrolide, clindamycin, fluoroquinolone, glycopeptide, trimethoprim/sulfamethoxazole, and antifungal agent.

^d ^Early treatment failure was defined as clinical deterioration within 72 h of treatment such as lack of response or worsening of fever, respiratory condition, and/or radiographic status requiring mechanical ventilation, aggressive fluid resuscitation or vasopressors, or death

HCAP healthcare-associated pneumonia

CAP community-acquired pneumonia

ICU intensive care unit

### Occurrence of PDR pathogens and clinical outcomes and in each severity class assessed by PSI

Table 4 shows the occurrence of PDR pathogens, early treatment failure, and mortality in each risk class. In low-risk patients, HCAP showed a higher occurrence of PDR pathogens (41.2% vs. 13.9%; *P *= 0.027) and early treatment failure (16.4% vs. 6.3%; *P *= 0.024). Moreover, patients with HCAP showed higher in-hospital mortality than those with CAP in the low (16.4% vs. 3.1%; *P *= 0.001) and intermediate (25.2% vs. 14.1%; *P *= 0.044) risk classes.

**Table 4:** Occurrence of PDR Pathogens and Clinical Outcomes and in Each Severity Class Assessed by the Pneumonia Severity Index in Patients

Pneumonia Severity Index Classes	HCAP (*n *= 231)	CAP (*n *= 296)	*P*-value
**PDR pathogens**^a^			
Low (I-III)	7/17 (41.2)	5/36 (13.9)	0.027
Intermediate (IV)	14/40 (35.0)	5/29 (17.2)	0.103
High (V)	8/22 (36.4)	4/18 (22.2)	0.332
**Early treatment failure**^b^			
Low (I-III)	9/55 (16.4)	10/159 (6.3)	0.024
Intermediate (IV)	21/115 (18.3)	18/99 (18.2)	0.988
High (V)	26/61 (42.6)	9/38 (23.7)	0.055
**In-hospital mortality**^b^			
Low (I-III)	9/55 (16.4)	5/159 (3.1)	0.001
Intermediate (IV)	29/115 (25.2)	14/99 (14.1)	0.044
High (V)	27/61 (44.3)	13/38 (34.2)	0.322

^a ^Number of patients/total number of patients whose causative pathogens were identified in each pneumonia severity index class (percentages).

^b ^Data are presented as number of patients/total number of patients in each pneumonia severity index class (percentages).

HCAP healthcare-associated pneumonia

CAP community-acquired pneumonia

PDR potentially drug-resistant

### Risk Factors for Occurrence of PDR Pathogens

The multivariate analysis of risk factors for the occurrence of PDR pathogens are shown in Table 5. Tube feeding and previous hospitalization within 90 days of pneumonia onset were significant risk factors; the corresponding odds ratios were 14.94 and 2.68. Of 162 patients with identified pathogens, 57 patients (35.2%) had been previously hospitalized within 90 days of infection. However, when previous hospitalization was classified into two different risk factors in relation to antibiotic treatment, 36 patients (63.2%) had previously been hospitalized with antibiotic treatment and 21 patients (36.8%) had been hospitalized without. The former was a significant risk factor (odds ratio = 2.45; 95% CI 1.19-5.02; *P *= 0.014), but the latter was not (odds ratio = 1.59; 95% CI 0.54-4.69; *P *= 0.398) (data not shown).

**Table 5:** Multivariate Analysis of Risk Factors for Occurrence of PDR Pathogens

Risk Factors	Odds Ratio	95% CI	*P-value*
Gender, female	1.50	0.75 - 3.01	0.256
Age	1.00	0.98 - 1.02	0.964
Tube feeding	14.94	4.62 - 48.31	<0.001
Residence in a nursing home or extended care facility	1.90	0.53 - 6.86	0.327
Intravenous chemotherapy within 30 days	0.62	0.22 - 1.77	0.373
Attended a hemodialysis clinic	2.81	0.86 - 9.19	0.087
Hospitalized in an acute care hospital for two or more days within 90 days of the infection	2.68	1.32 - 5.46	0.007

PDR potentially drug-resistant

CI confidential interval

## Discussion

This study showed that half of the hospitalized patients with pneumonia in a university tertiary referral hospital in South Korea were diagnosed with HCAP, and identified differences in comorbidities, pathogens, initial antibiotic regimens, and clinical outcomes between the HCAP and CAP groups. Moreover, tube feeding and prior hospitalization with antibiotic treatment within 90 days of pneumonia were found to be significant risk factors for PDR pathogens.

Patients with HCAP were more frequently classified into the intermediate- and high-risk classes than patients with CAP. More PDR pathogens were identified, more inappropriate antibiotic treatments were initiated, and clinical outcomes were worse for HCAP patients, especially those in the low and intermediate risk classes. The results of this study were consistent with those of previous studies reporting distinct clinical characteristics of HCAP and worse outcomes than CAP [10,12,14,24]. However, the baseline characteristics and the backgrounds of patients with HCAP differed slightly from previous reports. In this study, more patients with HCAP had malignancies (67.5%) and an immunosuppressive condition (61.5%) as comorbidity than other studies (14.2% to 22.3%) [12,24]. Furthermore, the HCAP group included a relatively lower proportion of patients residing in nursing homes or extended care facilities (9.1%) than previous reports (28.0% to 61.0%)[12,24]. These differences indicate the heterogeneous aspect of HCAP and the difficulty of establishing one unified approach for patients with HCAP [25].

Despite the high rate of anti-pseudomonal therapy in HCAP patients, a high proportion of inappropriate initial antibiotics were given to the patients in this study (37.0%), as compared with the studies of Shindo et al. (20.8%), Carratalà et al. (5.6%), and Park et al. (24.6%) [10,12,14]. This is likely due to the higher rate of PDR pathogen infection (36.7%), as compared with the aforementioned reports of Shindo et al. (22.1%), Carratalà et al. (3.5%), and Park et al. (29.3%) in HCAP patients, and the relatively high proportion of ESBL-producing Gram-negative pathogens [10,12,14]. In this study, *K. pneumoniae *(10.0%) was the most common pathogen in patients with HCAP, followed by *S. aureus *(5.6%), *S. pneumoniae *(4.8%), and *P. aeruginosa *(4.3%) in that order. In addition, the rate of ESBL-producing *K. pneumoniae *was relatively high in our study. This may be explained by differences in the underlying comorbidities of HCAP patients and their reasons for being in contact with the healthcare environment. A large proportion of patients with malignancies who had been regularly hospitalized for anti-cancer chemotherapy and a considerable proportion of patients with recent antibiotic therapy (42.0%) could explain an increasing colonization of ESBL-producing *K. pneumoniae*. In a report by Park et al., another study done in a tertiary hospital of South Korea, *K. pneumoniae *is also the second most common pathogen of HCAP and the rate of ESBL-producing *K. pneumoniae *comes to 79% in both CAP and HCAP [14]. Thus, efforts to identify the pathogens and to adjust empirical antibiotics accordingly, based on microbiological data, are more useful than automatic treatment with anti-pseudomonal broad-spectrum antibiotics.

Negative clinical outcomes, including early treatment failure and in-hospital mortality were all higher in HCAP patients than CAP patients. The differences were significant, especially among the low-risk class, and the occurrence of PDR pathogens was also more frequently observed in HCAP patients than in CAP patients among the low-risk class. These results were similar to the study of Shindo et al. in Japan, which showed higher mortality and PDR pathogens occurrence in HCAP patients than in CAP patients in the moderate severity class according to the A-DROP (age, dehydration, respiratory failure, orientation disturbance, and low blood pressure) scoring system [12]. In patients classified as high-risk, mortality was not different between HCAP and CAP patients, probably due to the severity of the illness itself, regardless of the presence of PDR pathogens. The poorer outcomes for patients with HCAP than for those with CAP in the low-risk class might be explained by the higher rate of early treatment failure (16.4% vs. 6.3%; *P *= 0.024), associated with a higher proportion of PDR pathogen (41.2% vs. 13.9%; *P *= 0.027), as shown in Table 4.

Although we could not find a significant difference in the rate of inappropriate initial antibiotics treatment between patients with HCAP and CAP, the proportion of inappropriate antibiotic treatment was significantly higher in HCAP patients infected with PDR pathogen than in those without (58.6% vs. 22.7%; *P *= 0.002) (data not shown), which is consistent with previous reports [14,26]. Therefore, it is important to identify risk factors for PDR pathogens in patients with HCAP to decide who should receive broad-spectrum antibiotics. These efforts would improve clinical outcomes and prevent the emergence of multi-drug resistant microorganisms from overuse of broad-spectrum antibiotics. According to multivariate analysis, significant risk factors for PDR pathogens included the use of antibiotics for more than two days during a prior hospitalization within 90 days of pneumonia onset as well as tube feeding. Thus, we suggest that physicians consider broad-spectrum antibiotics for treatment of HCAP patients with these risk factors for PDR pathogens.

HCAP is a newly defined group since 2005 and has been composed of heterogeneous patients with various severities of illness and different reasons for contact with the healthcare environment. Thus, there is little detailed data on these various HCAP groups, though it is associated with significant mortality and high health care costs [27,28]. This study may provide useful guidance in understanding the characteristics of HCAP and in developing therapeutic approaches for patients with HCAP in South Korea.

To fully appreciate our results, we should consider the limitations of the present study. First, this was a retrospective study in a single institution with a relatively short duration and may not represent South Korean medical institutions in general. However, this study shows the general characteristics of pneumonia patients admitted to tertiary hospitals in South Korea. Second, the etiology of pneumonia was identified in a low proportion of patients. Thus, the true incidence of PDR pathogen and its effects on the clinical outcomes could have been underestimated. However, 89% and 99% of the patients were evaluated using their sputum and blood samples, and our successful pathogen identification rate of 30% was not relatively low compared to the rate of 20-50% from previous prospectively designed studies [10,29,30]. Third, atypical pathogens could not be fully evaluated due to inadequate information in the medical records. Fourth, prior antibiotic use could not be fully estimated due to insufficient information from other clinics in the medical records.

## Conclusions

In summary, half of the hospitalized with pneumonia in a university tertiary referral hospital were diagnosed with HCAP. Patients with HCAP showed a higher occurrence of PDR pathogens, more frequent early treatment failure, and a higher mortality rate than patients with CAP, especially in patients with low-risk class. Those HCAP patients who underwent tube feeding and those who have been hospitalized and given antibiotic treatment within the previous 90 days should be mainly considered for broad-spectrum antibiotics.

## Abbreviations

HCAP: healthcare-associated pneumonia; CAP: community acquired pneumonia; PDR: potentially drug-resistant; PSI: pneumonia severity index; ATS: American Thoracic Society.

IDSA: Infectious Diseases Society of America; MRSA: methicillin-resistant *Staphylococcus aureus*; ESBL: extended spectrum *β*-lactamase; MAG: microparticle agglutination assay

SD: standard deviation; CI: confidence intervals; ICU: intensive care unit

## Competing interests

The authors declare that they have no competing interests.

## Authors' contributions

JJ carried out screening and acquisition of data, statistical analysis and participated in the writing of the manuscript. MP and YK carried out acquisition of data and statistical analysis. BP and SK participated in the study design and the analysis and interpretation of data. JC participated in the study design, analysis and interpretation of data and critical revision of the manuscript for important intellectual content. YK participated in the study design, analysis and interpretation of data and the writing of the manuscript. All authors have read and approved the final manuscript.

## Pre-publication history

The pre-publication history for this paper can be accessed here:

http://www.biomedcentral.com/1471-2334/11/61/prepub
